# Design & development of customizable web API for interoperability of antimicrobial resistance data

**DOI:** 10.1038/s41598-021-90601-z

**Published:** 2021-05-27

**Authors:** Jasleen Kaur, Jasmine Kaur, Shruti Kapoor, Harpreet Singh

**Affiliations:** 1 Division of Biomedical Informatics, Indian Council of Medical Research, New Delhi, 110029 India; 2 School of Computational and Integrative Sciences, Jawaharlal Nehru University, New Delhi, 110067 India; 3 Data Science Laboratory, Amity Institute of Integrative Science & Health, Amity University Gurgaon, Gurgaon, India

**Keywords:** Computational biology and bioinformatics, Software

## Abstract

Antimicrobial resistance (AMR) is a global health emergency. Complementary to developing new drugs, AMR can be monitored and controlled through cost-effective active surveillance of resistance. As an initiative to monitor resistance, countries all across the globe are collecting data using a variety of surveillance tools. Moreover, hospitals routinely collect the AMR data for treatment which is being stored in their Laboratory and Hospital Information systems (LIS-HIS). The generated clinical data is collected & stored in various formats, making it very difficult to analyze and generate national reports. To integrate the stored clinical data for predictive modeling and analysis, there is an immediate need for a one-stop data repository capable of importing and exporting data in simple data exchange formats (CSV/Excel). The paper highlights the design & development of *i*-DIA, a python-based web API to facilitate the interoperability of AMR data by automatically importing the bulk of medical data from CSV files into generic data management and analysis system. The *i*-DIA has been integrated and tested with the ICMR’s AMR surveillance network on in-house developed software, *i*-AMRSS. The *i*-AMRSS is presently collecting data from 31 laboratories across India and *i*-DIA has been used to import data generated from LIS & HIS of a few hospitals directly into the system. The paper also proposes the complete web-based framework (an extension of *i*-DIA) integrated with peer-to-peer system architecture.

## Introduction

The Antimicrobial resistance (AMR) is a global health emergency^1^. Decades of medical progress are under threat as our ability to treat infectious diseases reliably with antibiotics is compromised. Amidst a plethora of newer antimicrobials, resistance to even the most recently developed antimicrobials has been reported. In a recent report, it is estimated that approximately 0.7 million people die every year from drug-resistant strains of microbes. The number will increase to 10 million by 2050, surpassing cancer (8.2 million deaths per year)^2^.

The development of new drugs is one of the most coveted approaches to overcome the problem of drug resistance. A complementary and cost-effective approach is the judicious use of existing antimicrobials through better surveillance of resistance. As an effort towards monitoring AMR, countries have established surveillance networks and have developed tools (surveillance tools) for capturing AMR data. Based on their scope, these tools can be classified as collectors or integrators. The collectors are used by individual laboratories for monitoring AMR. For example, WHONET^3,4^, a tool that has been developed by WHO collaborating center and is presently endorsed by the WHO. It is an open-source windows-based software used worldwide by many laboratories for the management and analysis of microbiology data with a special focus on antimicrobial susceptibility test results. Other examples of laboratory-level surveillance include AMWeb^5^, a web-based tool launched by the Public Health England and British Society for Antimicrobial Chemotherapy. On the other hand, Integrators collect data from multiple laboratory-level surveillance systems. Examples include Japan Nosocomial Infections Surveillance (JANIS)^6^, The European Antimicrobial Resistance Surveillance Network (EARS-NET)^7^, and The National Antimicrobial Resistance Monitoring System (NARMS)^8^ from Japan, Europe, and the U.S.A respectively. All these tools are developed using different technologies and store data in different ways.

Indian Council of Medical Research (ICMR) has a well-established Antimicrobial Resistance Surveillance and Research Network (AMRSN)^9,10^ since 2013. The main aim of the ICMR AMRSN was to develop a hospital network to track the patterns in the antimicrobial susceptibility profile of medically significant human health-restricted bacteria and fungi. Since no previously available tools suited to the requirements of the network, the data management team at ICMR developed a web-based online AMR data entry system^11,12^, named *i*-AMRSS^23^ for capturing storage and analysis of AMR data. The software was launched in 2016 and is collecting data from 31 centers across India^11^. Some of these hospitals/centers are multispecialty sites with good LIS-HIS generating more than 500 patient records in a day. With a large patient burden and manual data entry, the data entry process has become a huge bottleneck for the data inflow.

Considering the diversity, there was a strong felt need for a platform-independent tool capable of integrating data from multiple sources. Thus, the concept of web API was initiated. The designed web API named *i*-DIA (Data Import App) has been integrated with *i*-AMRSS^23^ and tested for capturing excel based data from LIS and HIS of few hospitals in the *i*-AMRSS^11,12^.

This paper discusses the design and development of *i*-DIA and its piloting as a simplified and customized solution for the data import from the LIS and HIS into *i*-AMRSS^11,12,23^. The paper is structured as follows: Sect. 2 describes the methods that comprise the technology developed, research methodology followed, and schematic workflow of the targeted framework; Sect. 3 explicates the implementation results observed with the deployment of *i*-DIA into *i*-AMRSS^11,12,23^; Sect. 4 defines the conclusions and propose the extension of *i*-DIA to a complete web-based framework with a peer to peer system architecture.

## Methods

### Technology

The *i*-DIA^13,14^ is a platform-independent web-based API. It is developed as an open-source platform to provide a secure, highly configurable web-based framework, that incorporates the majority of functionality to import the customizable medical data (LIS and HIS) from CSV files to generic data management and analysis systems. Based upon the comprehensive analysis of the data from LIS and HIS of various hospitals in the network, it was concluded that the data is mainly exported in two formats: (a) the format with antibiotics names in rows (file comprises the antibiotic names and their corresponding susceptibility test values in the multiple respective rows) (supplementary Fig. 1a); (b) the format with antibiotic names as column headers (the file contains antibiotic names as multiple column headers and corresponding susceptibility test values are present in the respective rows) (supplementary Fig. 1b).

The current version of the *i*-DIA imports the data which is already pre-exported in CSV/excel sheet from the LIS and HIS of the hospitals enrolled in ICMR’s Antimicrobial Resistance Surveillance Network. The *i*-DIA accepts both the defined formats of pre-exported data and maps the required fields (headers, option sets, and antibiotic names) of the selected CSV/excel with the existing database fields of the generic data management and analysis system. The *i*-DIA generates the hospital-specific configuration files (JSON format) based upon the mapping. The generated JSON mapping can be used to import the bulk of medical data automatically from CSV/excel into the generic data management and analysis system. If the fields in the CSV/excel are the same, mapping is only a one-time process and the same mapping files can be used to upload multiple data files.

### Methodology

**Figure 1:**
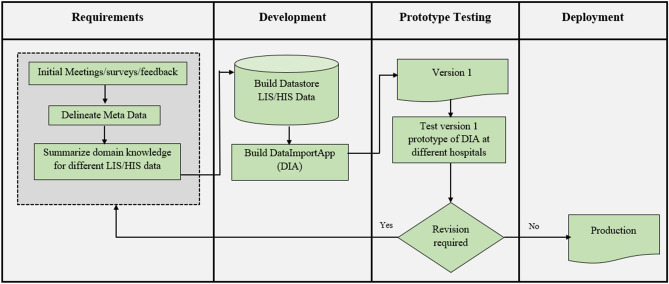
The research methodology followed for the design, development, and deployment of the *i-*DIA.

The research methodology followed in the design, development, and testing of the *i*-DIA incorporates different steps (see Fig. 1). The initial stage comprised of the surveys, meetings, feedback, and summarization of domain knowledge for different LIS and HIS data. The development stage included the building of the first version of the targeted *i*-DIA web API and the testing stage subsumes the testing of the *i*-DIA with the first use case of generic data management and analysis system (*i*-AMRSS). The testing phase imports the data which is already pre-exported in CSV/excel sheet from the LIS and HIS of the centers enrolled in ICMR’s Antimicrobial Resistance Surveillance Network.

### Architecture

The *i*-DIA is a Linux-based web API capable of interacting with any data management tool. The current version of *i*-DIA acts as an autonomous data import module. For testing, it has been integrated with ICMR’s ongoing Antimicrobial resistance surveillance system (*i*-AMRSS) where it could successfully import data from CSV/excel sheets into the *i*-AMRSS^23^ database. The complete system is hosted on Ubuntu 16.04 operating system using the apache web server. The design & development of *i*-DIA entail the use of different software tools and libraries such as Python programming language^15^; MySQL database^16^; Flask framework^17^; NumPy^18^ and Pandas^19^ library for data manipulation and import.

**Figure 2:**
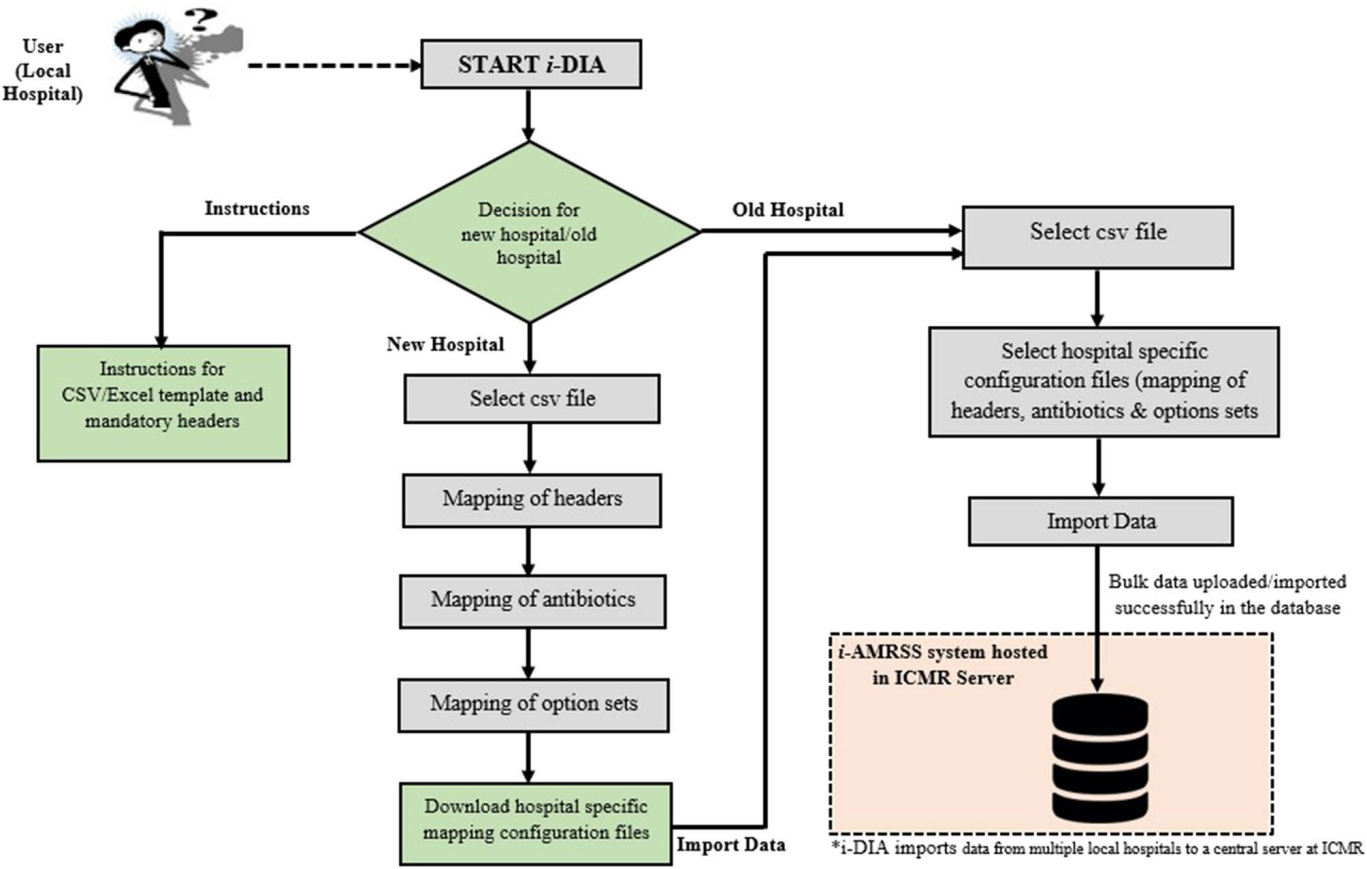
Schematic workflow of *i-*DIA.

The structural outline for the working of different modules of *i*-DIA is highlighted in Fig. 2. The new hospital module creates the hospital-specific mapping configuration files for the first time and saves the files with the hospital-specific unique id in the hospital network. The old hospital module considers that the hospital is already registered and mapping configuration files are already available in the registered hospital. The help/instruction module provides the basic instruction regarding the working of the *i*-DIA and mandatory headers of the *i-*AMRSS^23^ system.

### Data security and confidentiality

The *i*-DIA ensures data security through login-based access, where each user can only view the hospital-specific configuration files of their respective hospital. The sensitive patient identifiers such as phone number, address are not being captured in the *i*-AMRSS system^23^. Since the first version of *i*-DIA is integrated with the *i*-AMRSS system^23^, it will not import any sensitive patient identifiers. Only patient ID and sample ID are being captured which are encrypted in the *i*-AMRSS database, thus, ensuring patient confidentiality.

### Ethical approval

The present work described in the manuscript is a part of a larger antimicrobial resistance surveillance network (AMRSN) including thirty-one hospitals and a research initiative of the Indian Council of Medical Research (ICMR). These sites routinely share AMR data for clinical isolates received by clinical microbiology labs and capture antimicrobial prescriptions and clinical outcomes of patients with drug-resistant infections. This manuscript only describes a data import application (*i*-DIA) capable of transferring microbiology data from different hospitals connected in AMRSN to a central ICMR server. The *i*-DIA was piloted on ICMR's Antimicrobial Resistance Surveillance system (*i*-AMRSS). Each hospital of the *i*-AMRSS network has taken separate ethical clearance for the collection of AMR data. All the methods were carried out following relevant guidelines and regulations. The system does not require taking informed consent from all patients for which AMR data is added to *i*-AMRSS as (i)No patient is sampled exclusively for project work (ii)all samples are collected as part of the standard of care for patient management (iii)the data is utilized maintaining full confidentiality after removing all the patient identifiers. The study has been approved by the Ethics Committees of all the hospitals and patient data is managed following the Helsinki Declaration.

## Results

**Figure 3:**
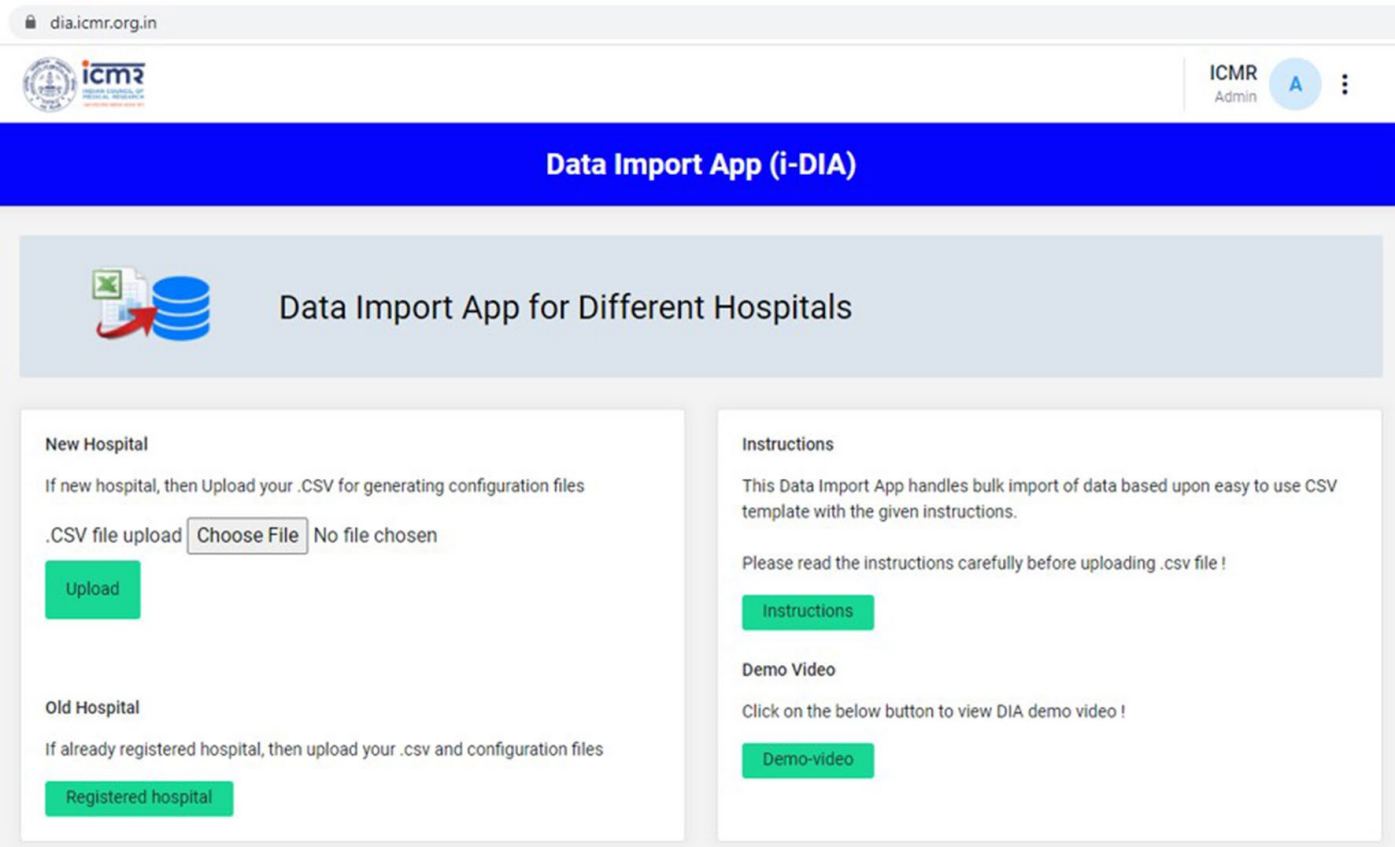
Home page of *i-*DIA.

The current version of the *i*-DIA module has been integrated with the *i*-AMRSS^23^ system. The bulk of data (LIS and HIS) can be transferred automatically from the local hospital (CSV/Excel format) to the generic data management and analysis system (*i*-AMRSS) based upon the hospital (hospitals that are enrolled in ICMR-AMR surveillance network) specific configuration file. The home page of the *i*-DIA web API consists of two modules: New Hospital Module and Old Hospital Module (see Fig. 3). The different steps followed in each module are implemented and tested for importing the sample test file (supplementary Fig. 6) into the *i*-AMRSS system^23^.

### New hospital module

**Figure 4:**
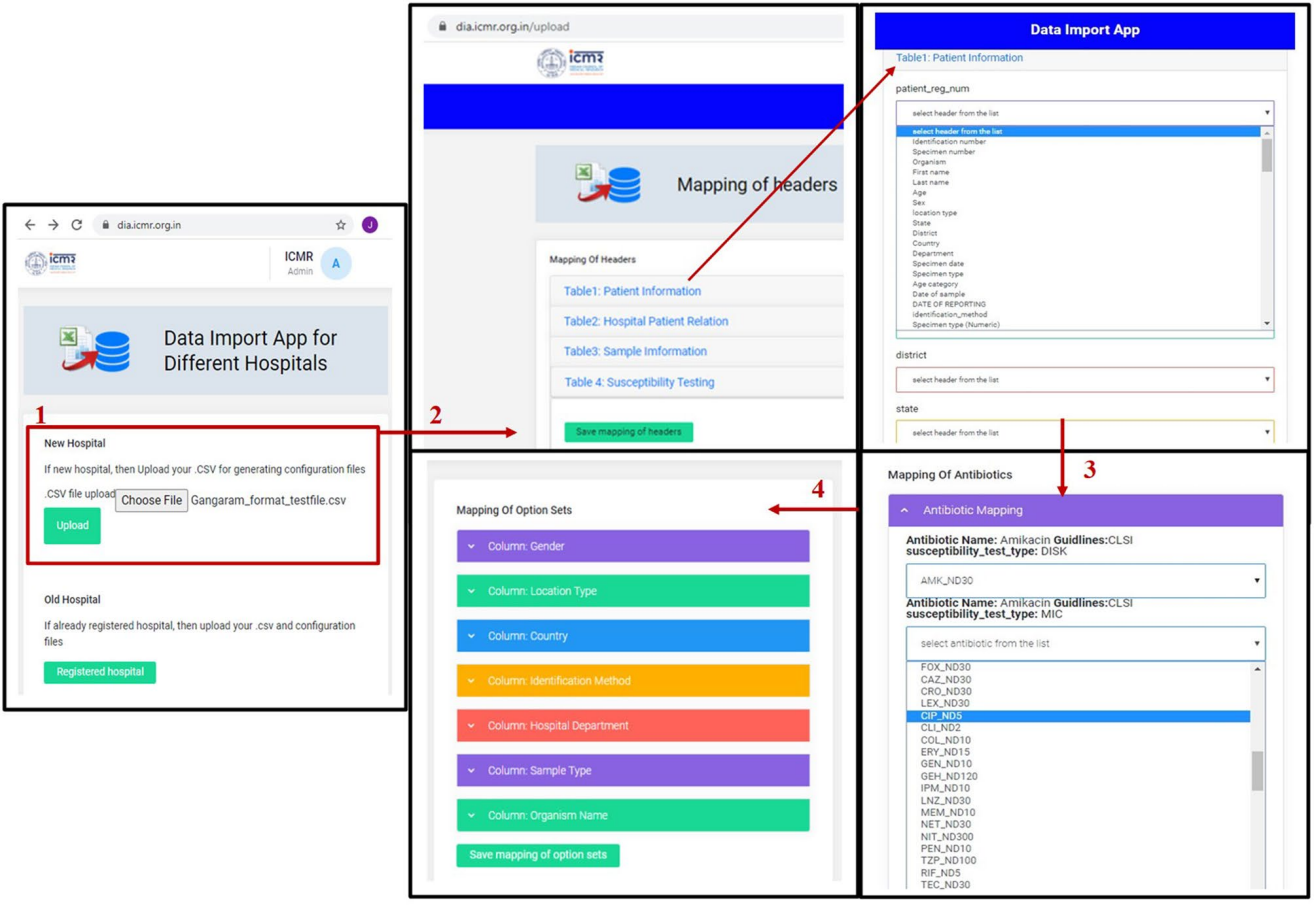
Different steps followed in the New Hospital Module of *i*-DIA.

The new hospital module provides an adaptable graphical user interface to create the hospital-specific configuration files. As shown in Fig. 4, different steps are followed in the new hospital module. The primary tables and their respective columns of the *i*-AMRSS system^23^ (Fig. 7) are shown in the new hospital module for the mapping of headers. In the first step, the module asks to select the desired hospital-specific CSV/excel file, followed by an option to customize the mapping of headers (supplementary Fig. 2a). The drop-down box comprising the list of headers from the selected CSV/excel file is given against each column of the *i-*AMRSS primary tables for the mapping of headers (supplementary Fig. 2b, c). Once the mapping of headers is done, the module saves the hospital-specific configuration file for the mapping of headers in the ICMR- AMR surveillance network prefixed with the unique id of the hospital.

If the mapping of the antibiotic header is not saved in the generated configuration file, then the module assumes that the selected CSV was in the second format and it automatically transposes the selected CSV file into the first format (the default format for the *i*-AMRSS system to accept the data).

As shown in Fig. 4(3), in step 3, the module asks to customize the mapping of antibiotics by their names. The drop-down box comprising the list of antibiotics from the selected CSV/excel is given against each antibiotic defined in the master table of the *i*-AMRSS database (supplementary Fig. 3).

The option sets are already defined against different columns of *i*-AMRSS primary tables. Thereof, in the fourth step, the option to customize the mapping of option sets is given (supplementary Fig. 4a,b), where the flexible interface is provided to map option sets of specific columns defined in the *i*-AMRSS primary tables with the option sets of respective headers given in selected CSV/excel (supplementary Fig. 4c–i).

**Figure 5:**
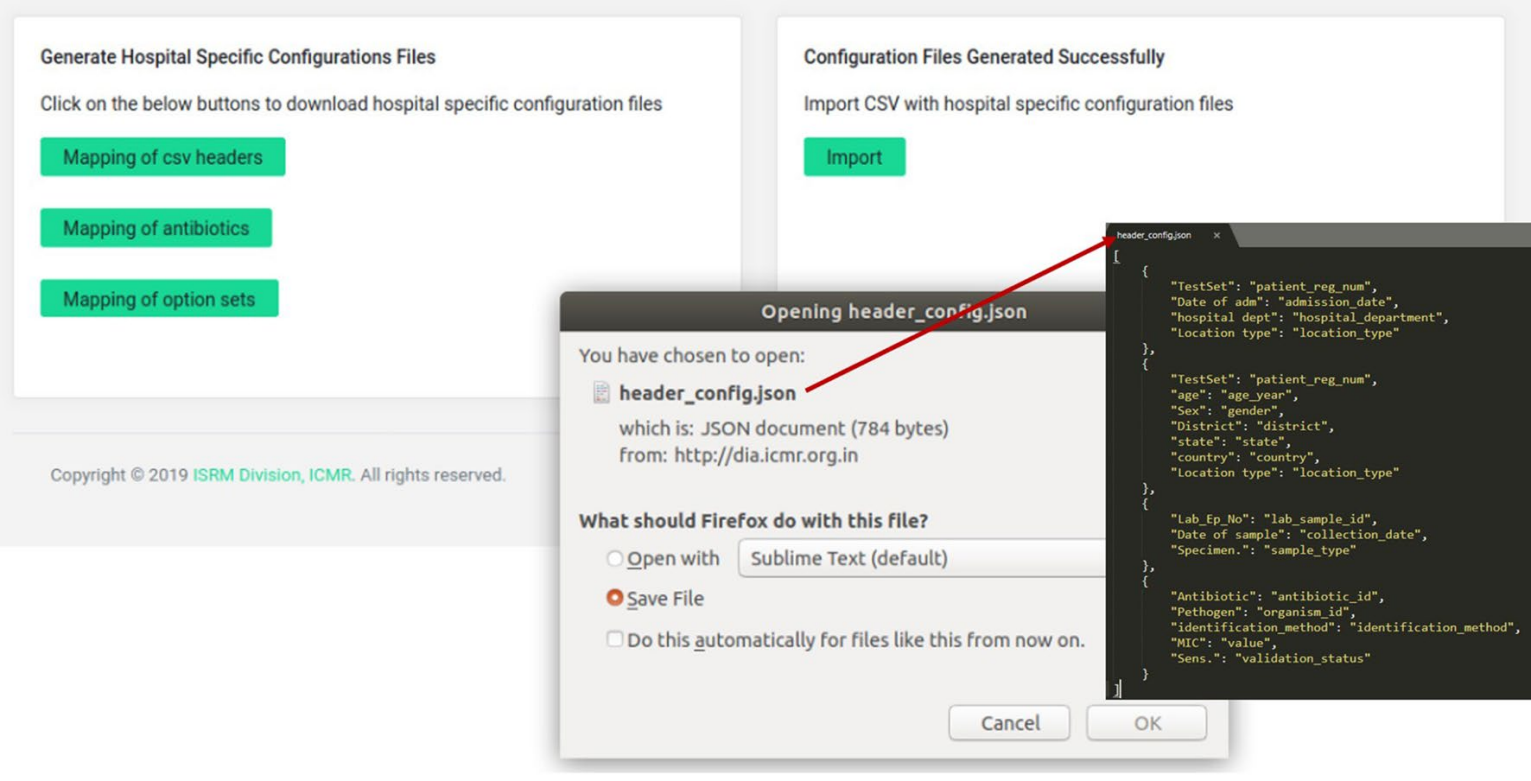
Hospital specific configuration files (JSON format).

The generated configuration files (in step2, step 3, and step 4) are saved in the ICMR- AMR surveillance network prefixed with the unique id of the hospital. Additionally, at this stage, the *i*-DIA asks to download all the hospital-specific configuration files on the local system for further use. The downloaded configuration file is in JSON format and includes hospital-specific mapping (see Fig. 5). The hospital is now considered as an old/already registered hospital and can transfer the complete excel/CSV file into the *i*-AMRSS system automatically through the import link or old hospital module.

### Old hospital module

**Figure 6:**
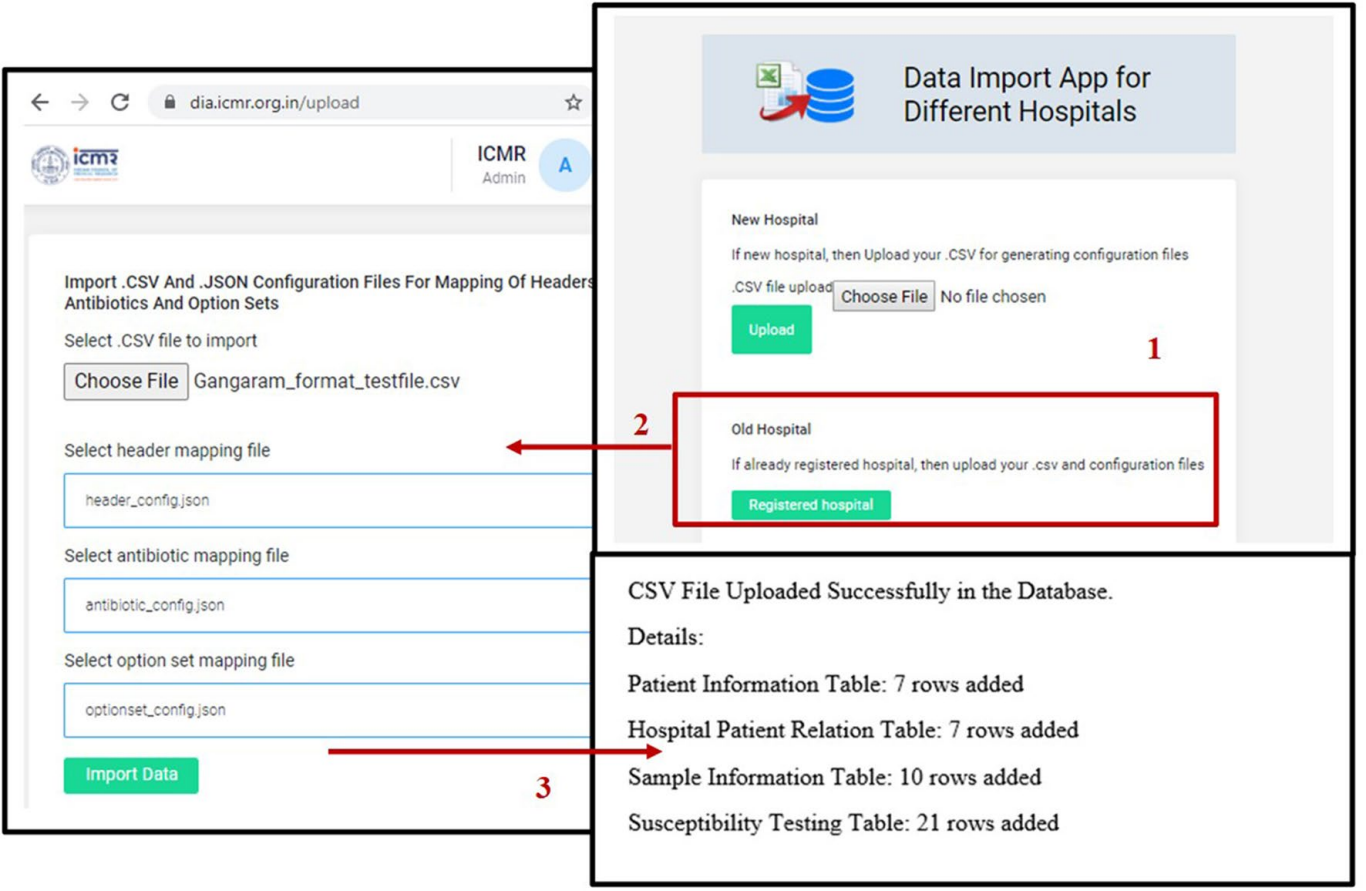
Different steps followed in the Old Hospital Module of *i*-DIA.

The old hospital module provides the adaptable graphical user interface to transfer the bulk of data (LIS and HIS) from the selected CSV/excel file into the *i*-AMRSS system^23^. Based upon the selected hospital-specific configuration files. As shown in Fig. 6, different steps are involved in importing the data through the old hospital module of the *i*-DIA. The old hospital module asks to select the CSV file, followed by an option to select the hospital-specific configuration files for mapping of headers, antibiotics, and option sets (supplementary Fig. 5).

**Figure 7:**
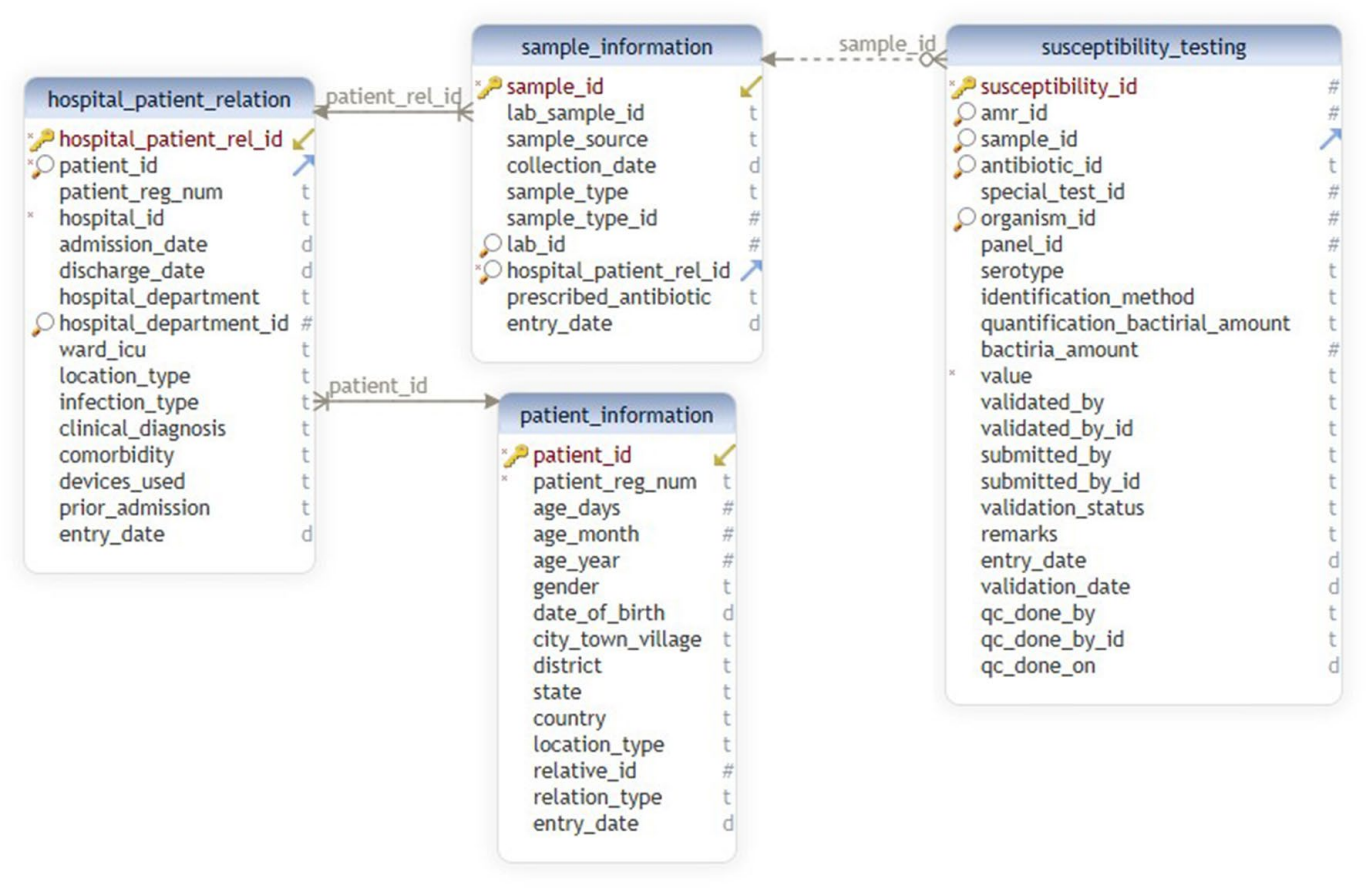
The Database structure of the primary tables of *i*-AMRSS^23^.

**Figure 8:**
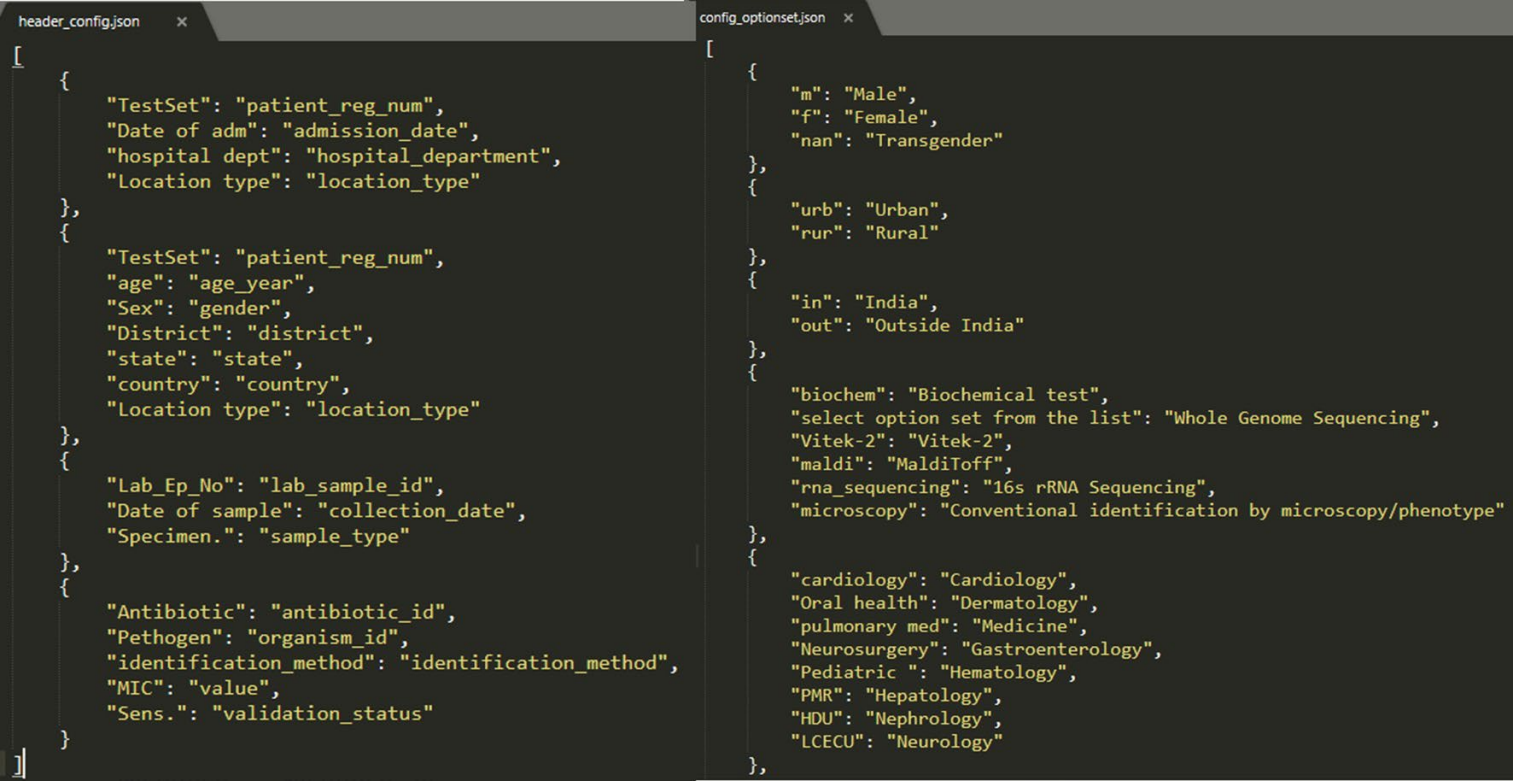
Hospital specific configuration files (header_config.json and config_optionset.json).

**Figure 9:**
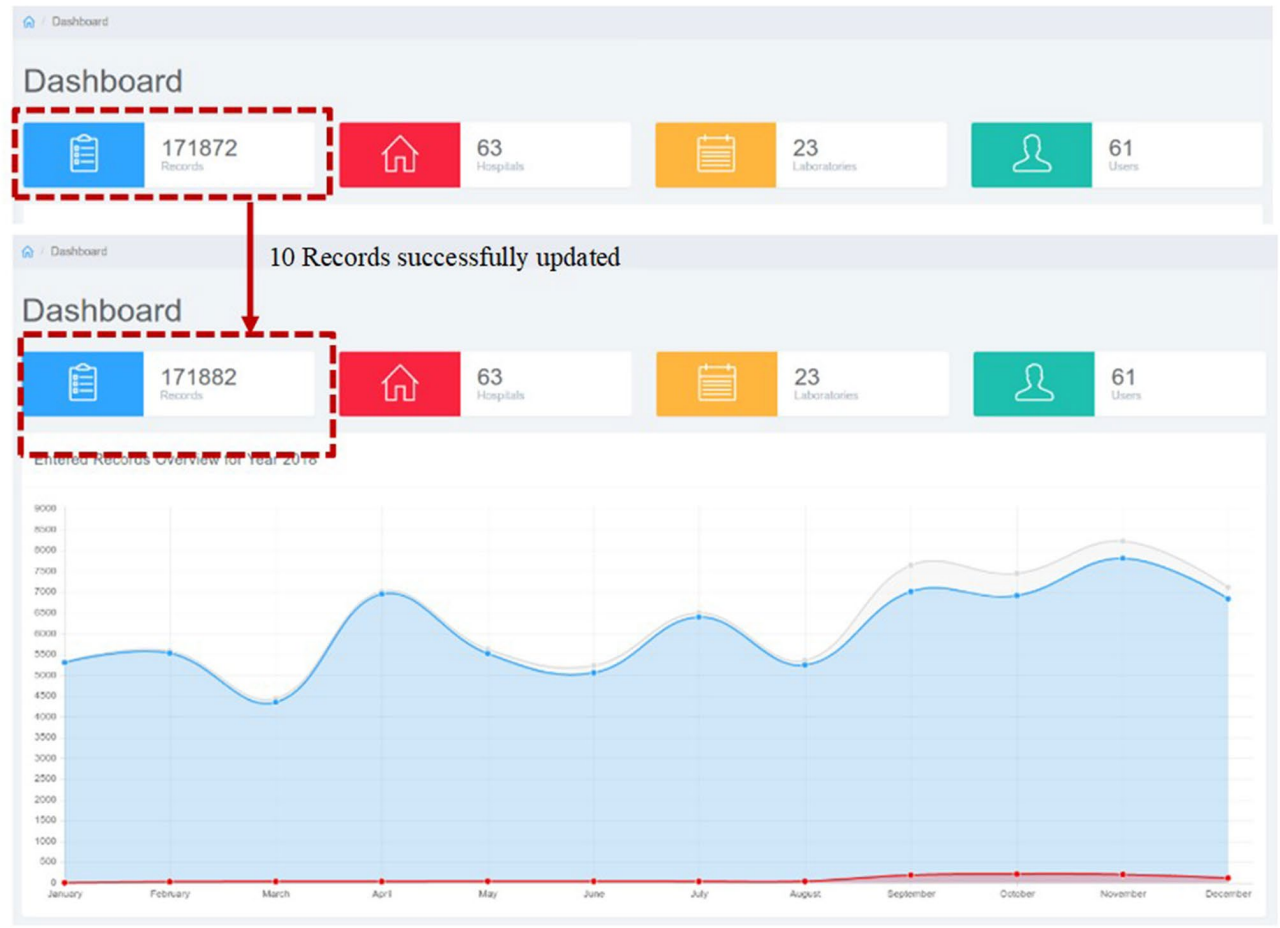
Updated Records.

For testing, the sample file (with 7 unique patient registration numbers and 10 unique lab sample IDs) (supplementary Fig. 6) has been used to import the data into generic data management and analysis system (*i*-AMRSS), based upon the selected hospital-specific configuration files. The main indices observed in a sample test file are Lab_Ep_No., TestSet, TestName, Pathogen, Antibiotic, MIC, Sens., Specimen, Sex, Country, Location type, Identification_method, hospital dept., DOB, Date of the sample, Date of adm, state, District, age. The database structure of the primary tables of the *i*-AMRSS system is shown in Fig. 7. The hospital-specific configuration files (headers, antibiotic and option set mapping files in JSON format) have been generated w.r.t sample test file data and the database structure of the primary tables of the *i*-AMRSS system. The generated JSON files for the sample test file have been shown in Fig. 8 in which the mapping has been shown for compulsory headers defined in the instructions (supplementary Fig. 2b). The sample test file gets successfully transferred into the *i*-AMRSS system (supplementary Fig. 7) and the new records are updated on the dashboard of *i*-AMRSS (see Fig. 9).

## Conclusions, discussion & future work

This paper proposes the platform-independent, user-friendly, open-source web-based framework *i*-DIA, to automatically import the bulk of medical data from CSV files into generic data management and analysis systems. To date, only GLASS^20^ is compiling data from various tools using BACKLINK^21,22^, a functionality in WHONET. However, BACKLINK is WHONET based and can only be used on Windows systems. The *i*-DIA is a platform-independent, web-based, and login-protected tool.

Some of the unique features of *i*-DIA include: (1) providing the freedom to upload the data in three dimensions- customizable mapping of headers, antibiotics, and option sets; (2) providing an easy to use graphical user interface for multiple users; (3) adopting the new methodology of importing the bulk of medical data from CSV file into the *i*-AMRSS based upon the hospital-specific configuration files.

**Figure 10:**
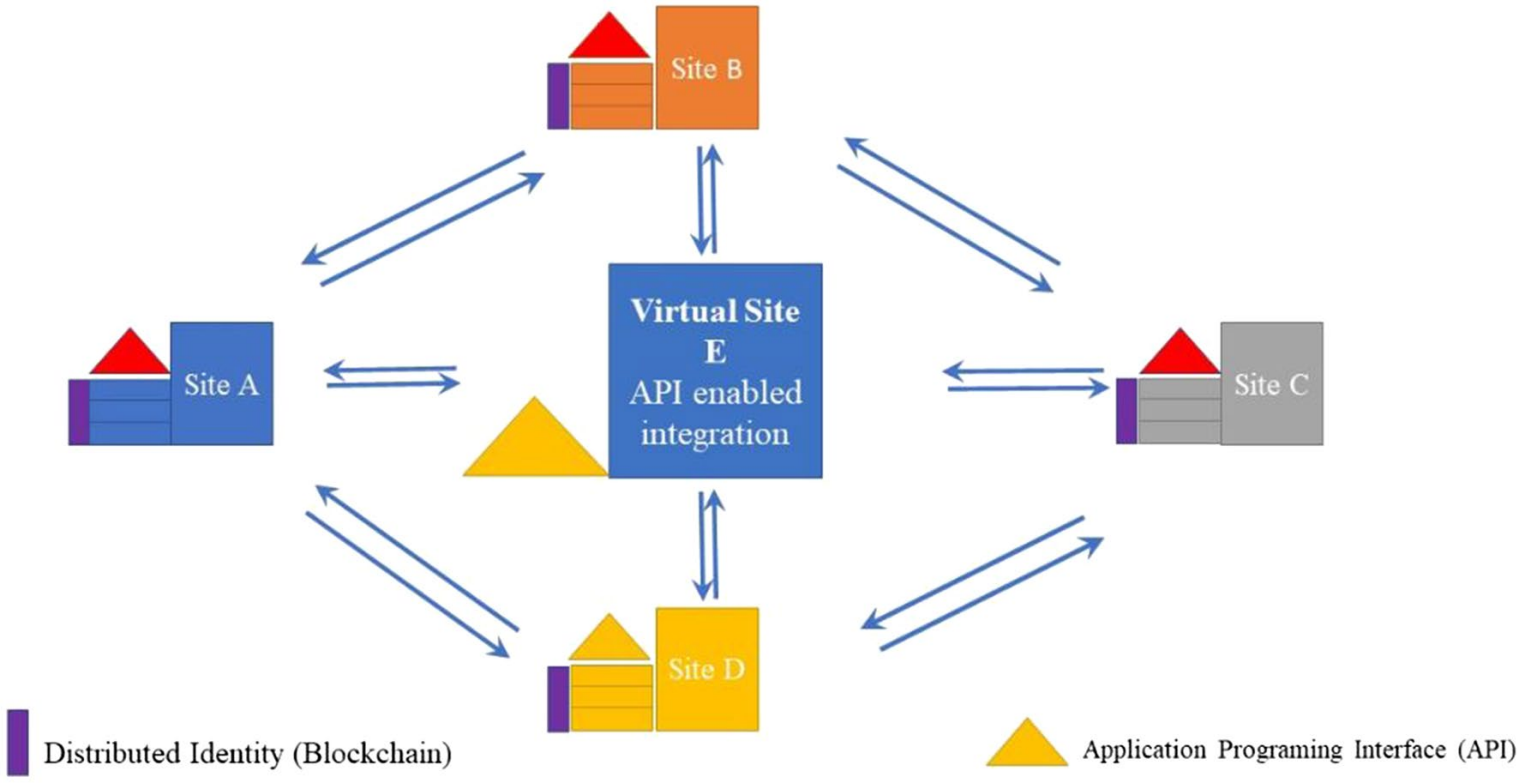
The architecture of the proposed web-based framework based upon the peer to peer network.

The *i*-DIA is intended for extension in terms of a complete web-based framework designed with peer-to-peer system architecture. It has been proposed to automatically import the bulk of medical data from CSV files into generic data management and analysis systems in a peer-to-peer network. The proposed complete web-based framework (an extension of *i*-DIA) provides the new technology to import the bulk of medical data automatically in the specific data management and analysis system integrated with peer to peer network (see Fig. 10). The proposed targeted framework includes the (1) integration of distinct Data Import web API (*i*-DIA) at the local sites for the local data entry and storage based upon the hospital-specific configuration files; (2) integration of the blockchain decentralized identity of the respective local site to ensure the privacy and trust between multiple local sites.

Future work will also focus on the following (1) design and development of the generic *i*-DIA that can be integrated with different data management and analysis systems, other than *i*-AMRSS; (2) The complete *i*-DIA is targeted to be an open-source tool, which can be customized by the users as per the requirement of the specific data management system; (3) develop the means for the registered hospitals in the AMRSN network to interact with the instructors regarding the working of the complete *i*-DIA web API; (4) the legal framework that will allow the hospitals in the network to upload the identifiable data (HIS and LIS) to the central server.

## Supplementary Information

Supplementary Information.
