# Supplementary material for: Design & development of customizable web API for interoperability of antimicrobial resistance data
Source: Sci Rep. 2021 May 27;11:11226. doi: 10.1038/s41598-021-90601-z (PMC8160260; doi:10.1038/s41598-021-90601-z)

**SUPPLEMENTARY FIGURES**

**Design & Development of Customizable Web API for Interoperability of Antimicrobial Resistance data**

Jasleen Kaur^1^ , Jasmine Kaur^1,2,3^ , Shruti Kapoor^1^ , and Harpreet Singh^1^*

^1^ Division of Biomedical Informatics, Indian Council of Medical Research, New Delhi 110029, India

^2^ School of Computational & Integrative Sciences, Jawaharlal Nehru University, New Delhi, 110067, India

^3^ Data Science Laboratory, Amity Institute of Integrative Science & Health, Amity University Gurgaon.

*Correspondence Address:

Harpreet Singh

Email: hsingh@bmi.icmr.org.in

Division of Biomedical Informatics, Indian Council of Medical Research, New Delhi 110029, India

**Supplementary Figure 1: Different formats of data**

1. first format with antibiotics names in rows
2. second format with antibiotic names as column header


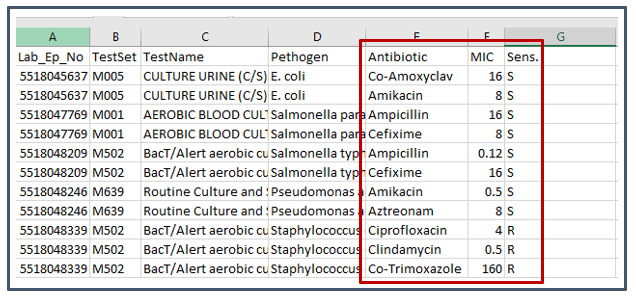


**1 (a)**


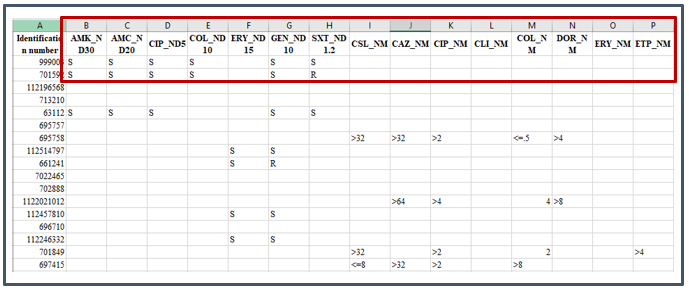


**1 (b)**

**Supplementary Figure 2: Mapping of Headers**

Manual data entry in generic data management and analysis system (*i*-AMRSS) involves saving the data into four tables of *i-*AMRSS database (Patient Information, Hospital Patient Relation, Sample Information and Susceptibility Testing). Therefore, mapping of headers is done with respect to four different tables.

1. WebAPI showing four different tables.
2. Mandatory headers of respective tables that are required to map.
3. Sample mapping of patient information table.
4. Sample mapping of susceptibility testing table.


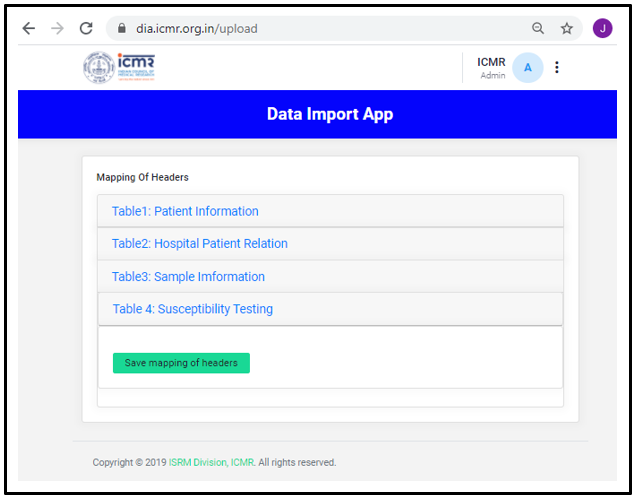


**2 (a)**


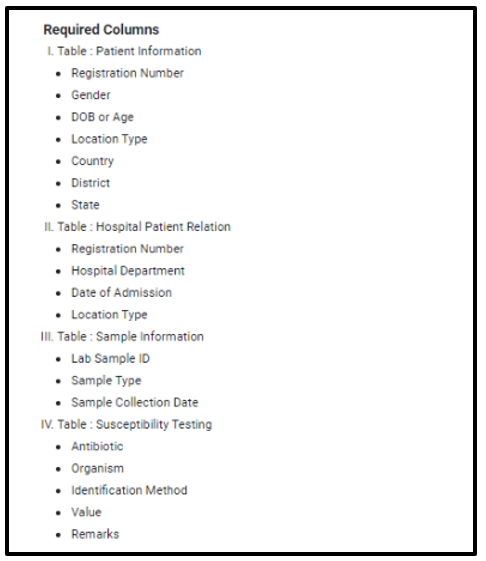


**2 (b)**


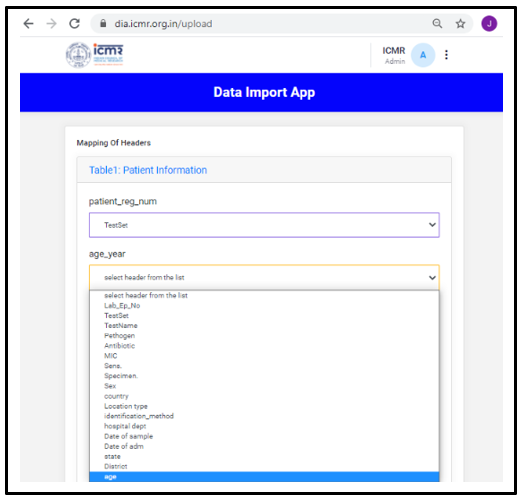


**2 (c)**


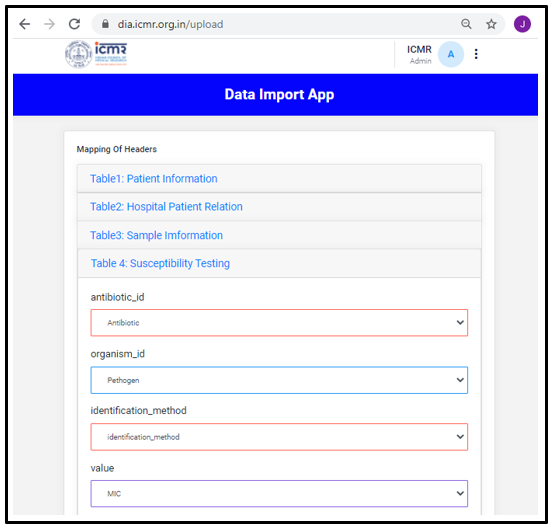


**2 (d)**

**Supplementary Figure 3: Mapping of Antibiotics**

Manual data entry in generic data management and analysis system (i-AMRSS) involves saving the antibiotic id against each defined antibiotic name in the antibiotic table of *i*-AMRSS system.

1. Antibiotic table of *i*-AMRSS system, showing antibiotic names based upon the guidelines and susceptibility test type.


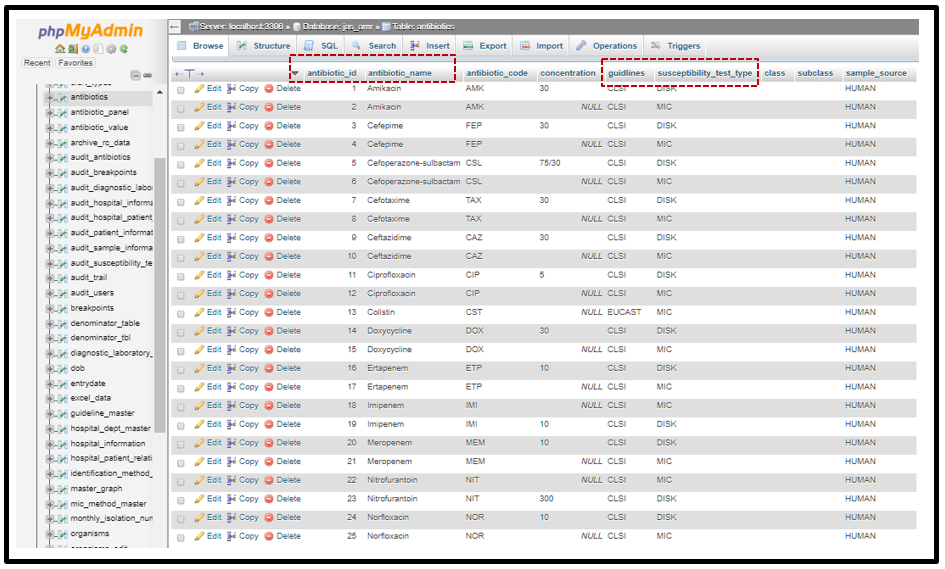


**3 (a)**

1. The name given in the first row is the name of the antibiotic saved in the database with the CLSI/EUCAST guidelines and DISK/MIC susceptibility test type and the drop-down list given in the second row comprises the antibiotic names abbreviated in the uploaded csv/excel file.


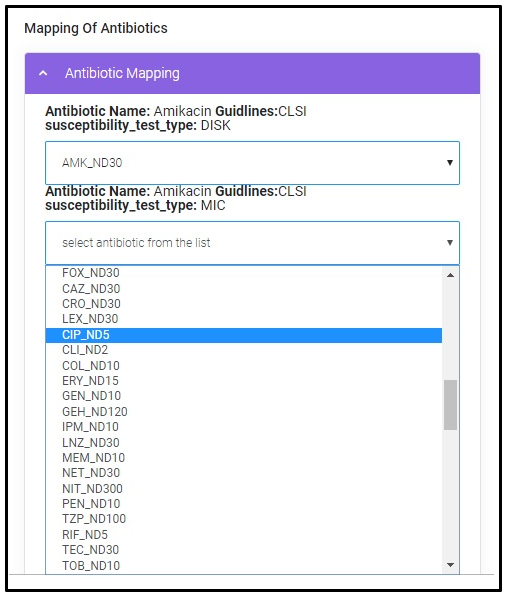


**3 (b)**

**Supplementary Figure 4: Mapping of option sets**

Manual data entry in generic data management and analysis system (*i*-AMRSS) involves option sets for 7 different headers/columns (Gender, Location Type, Country, Identification Method, Hospital Department, Sample Type and Organism Name). Therefore, mapping of option sets is done with respect to option sets given in the database against defined 7 different headers/columns.

1. WebAPI showing seven different headers/column for option set mapping.
2. Mobile view of the option set mapping
3. Option set mapping of ‘Gender’ column (e.g. here Male, Female and Transgender are the options defined in the ‘Gender’ option set of *i*-AMRSS system whereas m, f and nan are given as options in uploaded sample file).
4. Option set mapping of ‘Location Type’ column.
5. Option set mapping of ‘Country’ column.
6. Option set mapping of ‘Identification Method’ column.
7. Option set mapping of ‘Hospital Department’ column.
8. Option set mapping of ‘Sample Type’ column.
9. Option set mapping of ‘Organism Name’ column.


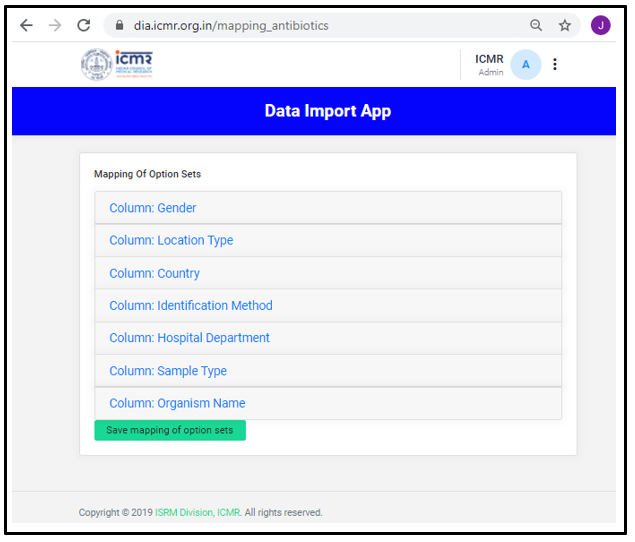


**4 (a)**


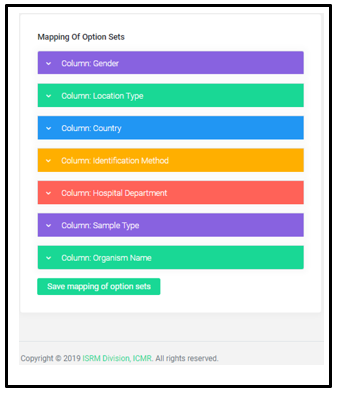


**4 (b)**


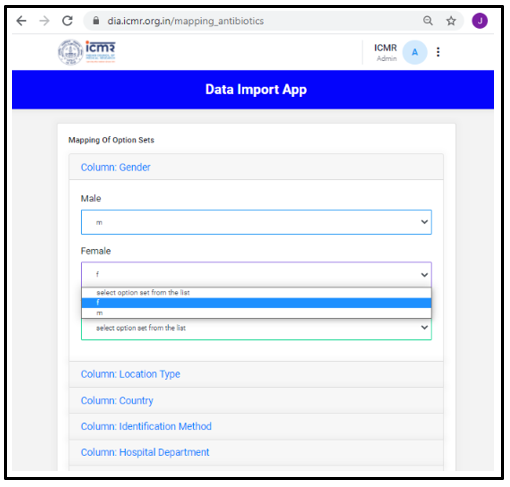


**4 (c)**


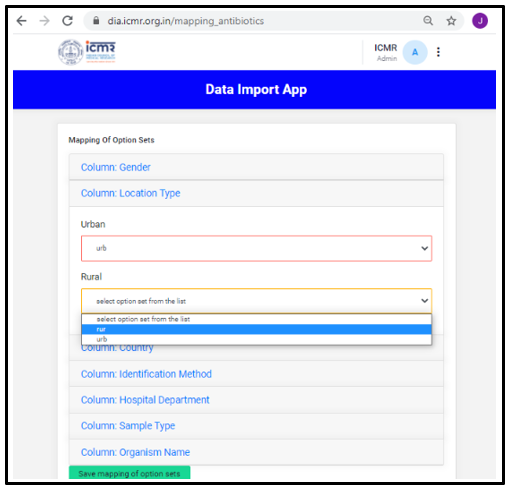


**4 (d)**


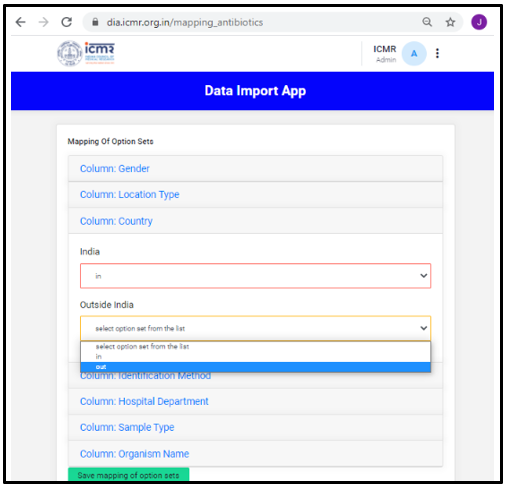


**4 (e)**


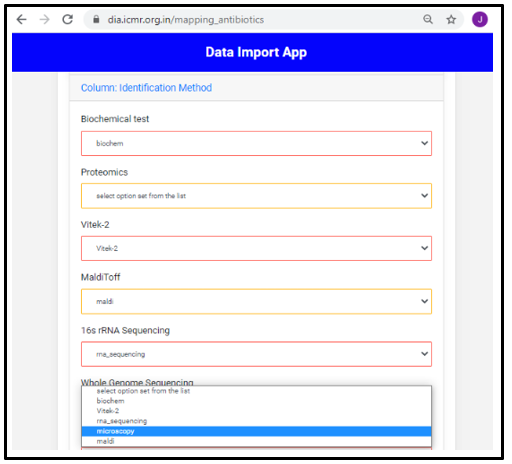


**4 (f)**


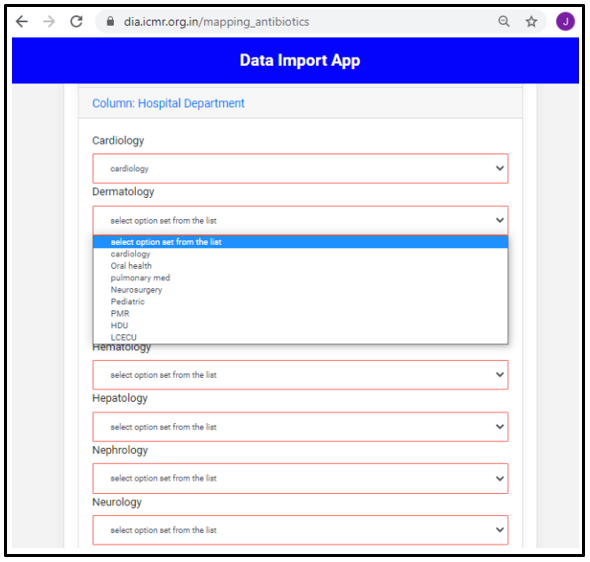


**4 (g)**


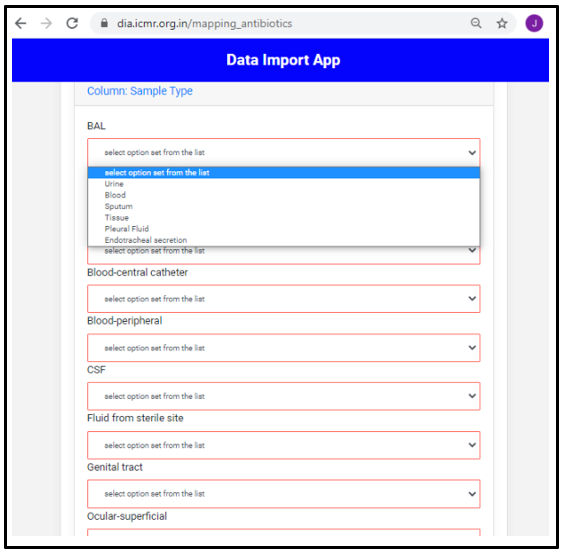


**4 (h)**


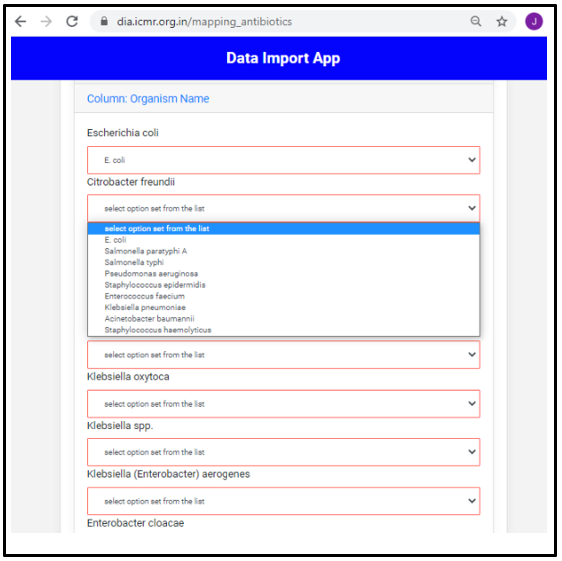


**4 (i)**

**Supplementary Figure 5: Import Data**

WebAPI showing an option to select csv/excel file and select hospital specific configuration files (in case of registered hospital) from the drop-down list.


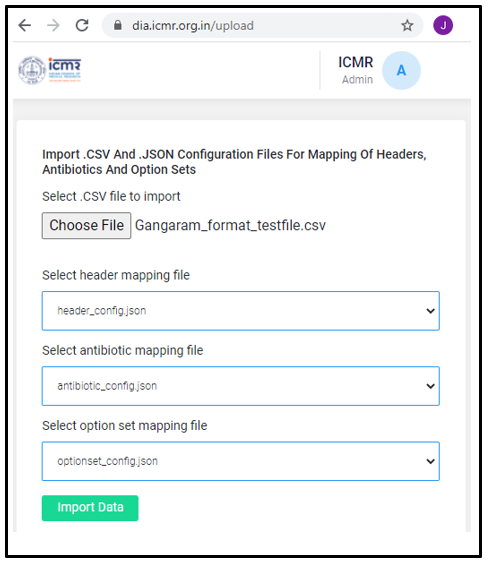


**Supplementary Figure 6: Sample file used**

1. First format (consists of antibiotic header that comprises the antibiotic names and their corresponding susceptibility test values in the multiple respective rows) (supplementary figure 1(a)).
2. 7 unique patient registration number.
3. 10 unique Lab Sample ID


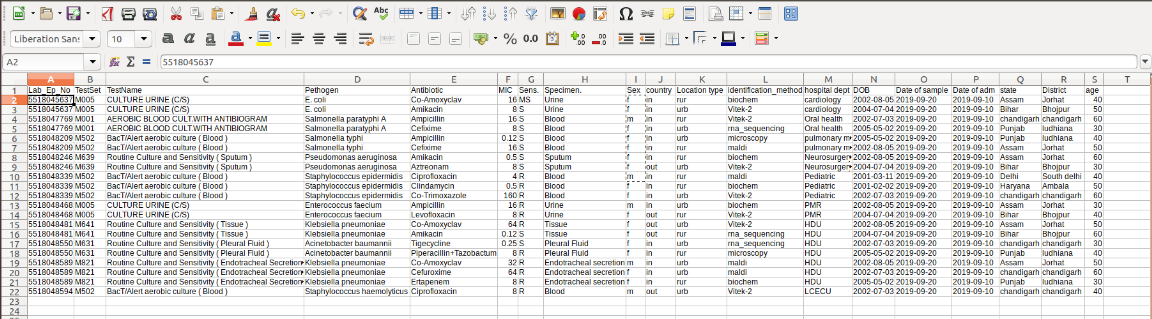


**Supplementary Figure 7: Data Imported Successfully**

1. WebAPI showing that the data has been successfully imported into database of the *i*-AMRSS system and details of the updated records for each table are shown.
2. In patient information table and hospital patient relation table, 7 rows are added as there are 7 unique patient registration numbers in the selected sample file (figure 10).
3. In sample information table, 10 rows are added, as there are 10 unique sample ID’s in the selected sample file (supplementary figure 6).
4. In susceptibility testing table, 21 rows are added, as there total 21 positive rows in the selected sample file (supplementary figure 6).


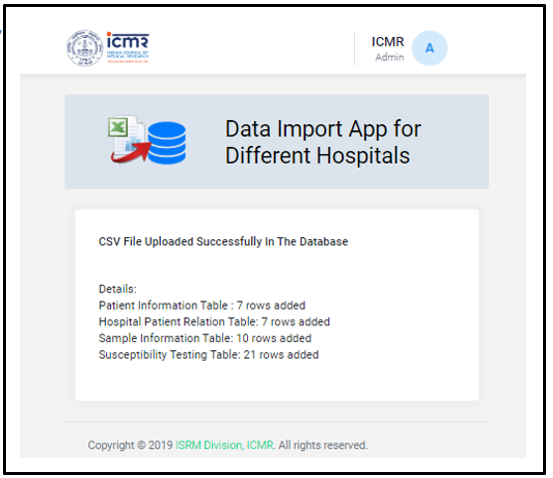

Supplement: Supplementary file 1 — Supplementary Information. [file 41598_2021_90601_MOESM1_ESM.docx]
